# Diagnostic model of saliva peptide finger print analysis of primary Sjögren's syndrome patients by using weak cation exchange magnetic beads

**DOI:** 10.1042/BSR20130022

**Published:** 2013-07-16

**Authors:** Pan Wei, Winston Patrick Kuo, Feng Chen, Hong Hua

**Affiliations:** *Department of Oral Medicine and Traditional Chinese Medicine, School of Stomatology, Peking University, Beijing 100081, People's Republic of China; †Harvard Catalyst Laboratory for Innovative Translational Technologies, Harvard Medical School, Boston, MA, U.S.A.; ‡Central Laboratory, School of Stomatology, Peking University, Beijing 100081, People's Republic of China

**Keywords:** mass spectrum, peptidomics, salivary diagnostics, Sjogren’s syndrome (SS), weak cation exchange beads, MALDI-TOF-MS, matrix-assisted laser-desorption ionization-time-of-flight MS, PRP, proline-rich protein, pSS, primary Sjögren’s syndrome, sSS, secondary SS, WS, whole saliva, WCX, weak cation exchange

## Abstract

Saliva diagnostics has become an attractive field utilizing nanotechnology and molecular technologies for pSS (primary Sjögren's syndrome). However, no specific methods have been established. To refine the diagnostic power of the saliva peptide finger print for the early detection of pSS, we screened the expression spectrum of salivary peptides in pSS patients by using mass spectrometry MALDI-TOF-MS (matrix-assisted laser-desorption ionization-time-of-flight MS) combined with magnetic bead. The present study was comprised 12 pSS patients and 13 healthy controls and broken down to two different phases. In the initial ‘exploratory phase’, we enrolled seven pSS patients with eight age- and sex-matched healthy volunteers. Proteomics analysis of the unstimulated salivary samples was conducted to generate proportional peptide mass fingerprints. A diagnostic model was established. The testing cohort of the second ‘validation phase’ was represented by five pSS patients and five age- and sex-matched healthy controls. The diagnostic power of this diagnostic panel was then validated. The results showed seven m/z (mass-to-charge) ratio peaks with significant differences. Five peptides were up-regulated and two down-regulated in the pSS patients compared with matched healthy subjects. In the validation phase, four out of five pSS patients were diagnosed as pSS, and four of the five healthy controls were diagnosed as healthy controls, respectively. Potential biomarkers were also primarily predicted. The novel diagnostic proteomic model with m/z peaks 1068.1 Da, 1196.2 Da, 1738.4 Da, 3375.3 Da, 3429.3 Da, 3449.7 Da and 3490.6 Da is of certain value for early diagnosis of pSS.

## INTRODUCTION

SS (Sjögren's syndrome) is a systemic autoimmune disease characterized by lymphocytic infiltration and destruction of the exocrine glands resulting in significant reduction of saliva and tear production [1]. The disease may be presented as either pSS (primary Sjögren's syndrome) or sSS (secondary Sjögren's syndrome). sSS has been associated with other autoimmune diseases such as RA (rheumatoid arthritis) and SLE (systemic lupus erythematosus). pSS predominantly affects women, who are nine times more likely to be affected. The prevalence of pSS is 0.4% in Caucasian women [2] and 0.33–0.77% in Chinese women [3]. Although many serologic components have been found to be involved in the disease course, including rheumatoid factor, antinuclear antibodies and antibodies to extractable cellular antigens Ro/SS-A and La/SS-B, no pathognomonic biomarkers for this disease have yet been found.

There are several sets of diagnostic criteria for pSS. Currently, the diagnosis of pSS is based on the American–European Consensus Group Revised Classification Criteria, which was made public in 2002 [4]. The Revised Criteria encompass the presence of subjective and objective sicca manifestations, autoantibodies to Ro/SS-A and La/SS-B antigens, and characteristic histopathological findings in the minor salivary glands with an average of 50 or more lymphocytes (focus) per 4 mm^2^ of minor salivary gland tissue. Nonetheless, owing to the complexity of the diagnostic process and the lack of biomarkers that are both sensitive and specific, diagnosis of pSS usually lags behind symptom onset by years. Biopsy of the minor salivary gland and parotid gland is highly specific for the diagnosis of pSS, but the procedure is invasive, requires evaluation by an expert histopathologist, and may have irreversible sequelae [5]. Owing to the invasiveness of the approach, many patients are reluctant to undergo this diagnostic method. On the other hand, it is crucial to diagnose pSS in its early stages to avoid destructive processes that will lead to a poor quality of life and early invalidity. The development of a comparatively accurate and non-invasive method for detecting pSS, especially in the early stages, would be invaluable.

In order to improve pSS diagnostic methods, during the last few years, there has been increasing interest in salivary proteomics as a promising tool to identify new salivary biomarkers that may allow non-invasive diagnosis [6–12]. Saliva is a complex fluid composed of a variety of electrolytes, metabolites, nucleotides, polynucleotides and proteins, which is secreted from the salivary gland. As it may closely reflect underlying pathogenic processes in the salivary gland, analysis of saliva from pSS patients is viewed as a promising method of diagnosing pSS. In addition, diagnosis based on the saliva test provides several key advantages, such as low cost, non-invasiveness and simple sample collection and processing. The composition of saliva in pSS patients has been found to be significantly different from that in healthy subjects in several studies [6,8–11]. However, the components of saliva are far from being completely defined, especially its low molecular weight components, such as PRPs (proline-rich proteins), statherins, histatins and cystatins, as well as defensins, which are immunopeptides of epithelial and neutrophilic origin [7]. The molecular weight of most of these proteins is below 10 kDa. To-date, very few studies have concentrated on low-molecular-weight salivary proteins (<10 kDa) and peptide profiles in pSS patients and healthy subjects.

In the present study, we investigated differences in the salivary peptide (1–10 kDa) profiles of pSS patients and healthy subjects. We also found a panel of salivary peptides that are of certain value to diagnose pSS based on a comparison with healthy subjects.

## MATERIALS AND METHODS

### Ethics statement

This study was approved by the Peking University Biomedical Ethics Committee. Adult subjects and parents of pediatric subjects signed an informed consent form before the start of research.

### Patients and saliva collection

All patients were enrolled at the department of Oral Medicine and Traditional Chinese Medicine, Peking University School and Hospital of Stomatology between November 2011 and January 2012. Samples of unstimulated WS (whole saliva) were collected from 12 pSS patients and 13 healthy controls for comparative analysis. The 12 pSS patients involved in this study were all diagnosed according to the revised international classification criteria for SS [4], with a mean age of 53.9±12.26 (range 23–70) years, and all of them had not taken any prescription medications, which would alter flow rates by the time of saliva collection. The patients with other medical disorders and complicated medication conditions were excluded from this study. Detailed clinical and serological characteristics of pSS patients were showed in Table 1. A total of 13 WS samples were collected from healthy controls with a mean age of 46.9±1.66 (range 45–49) years. All of the participants are female. To reduce the possible influence of seasonal and circadian variations on the salivary flow rate, all samples were taken between 9:00 and 11:00 am. All patients and healthy controls refrained from eating, drinking or chewing gum for at least 90 min before sampling, and all samples were taken by the same dentist. Before collection, the subject was instructed to rinse orally with water and then rest for 5 min with the eyes open and head tilted slightly forward. The WS was collected over a period of 15 min or more with a paper cup on ice, and then centrifuged at 2600 ***g*** for 15 min at 4°C. The supernatant was then removed and immediately stored at −80°C in 200 μl aliquots for further analysis. Prior to proportional peptide mass fingerprint analysis, the deep-frozen samples were thawed quickly by immersion in a hot water bath for a short period of time, in order to maintain protein integrity.

**Table 1:** Detailed clinical and serological characteristics of pSS patients pSS, primary Sjögren's syndrome; ND, not detected.

Patient	Dry mouth	Dry eye	Unstimulated WS flow rate*	Parotid Sialography*	Schirmer*	Anti-Ro	Anti-La	Biopsy*	Other connective tissue disease
pSS#1	+	+	+	+	+	+	−	ND	−
pSS#2	+	+	+	+	+	+	+	ND	−
pSS#3	+	−	+	+	+	+	−	ND	−
pSS#4	+	+	+	+	+	+	−	+	−
pSS#5	+	+	+	+	ND	+	+	ND	−
pSS#6	+	−	+	+	+	−	−	+	−
pSS#7	+	+	+	+	+	+	−	ND	−
pSS#8	+	+	+	ND	+	+	−	ND	−
pSS#9	+	+	+	+	+	+	+	ND	−
pSS#10	+	+	+	+	+	+	−	+	−
pSS#11	+	+	+	+	+	+	−	ND	−
pSS#12	+	+	+	+	ND	+	−	ND	−

*The criteria for positive results were based on the American–European Consensus Group Revised Classification Criteria for SS.

### Instruments and reagents

A WCX (weak cation exchange) magnetic bead kit (Bioyong SPE-C) and robotic separation device for a 96-well plate format magnetic separator were purchased from Boiyong Company. LT-2 MALDI-TOF-MS (matrix-assisted laser-desorption ionization-time-of-flight MS) was used for the MS analysis. Mass concentration (0.3 g/l) and ethanol (chromatographic grade)/acetone (chromatographic grade)=2/1 were prepared freshly.

### Sample application and MALDI-TOF-MS analysis

The suspension in the WCX magnetic bead kit was mixed by shaking. After eluting and beating, the magnetic beads were separated from the protein, and the eluted peptide samples were transferred to a 0.5-ml clean sample tube for further MS analysis. Five microlitres of HCCA substrate solution (0.4 g/l, dissolved in acetone and ethanol) and 0.8–1.2 μl of elution were mixed. Then, 0.8–1.2 μl of this mixture was applied to a metal target plate and dried at room temperature. Finally, the prepared sample was analysed by MALDI-TOF MS. A range of 1000–10000 Da peptide molecular weight was collected, and 400 shots of laser energy were used. Peptide mass fingerprints were obtained by accumulating 50 single MS signal scans. The saliva samples collected from each patient were analysed serially for three times by using MALDI-TOF-MS. The mean values of each sample were used for data analysis.

### Statistical analysis

The *t* test was used for comparison between the two groups. Data were analysed using the BioExplorer statistical package (BioyongTech). A *P*-value <0.05 was considered statistically significant.

## RESULTS

**Figure 1:**
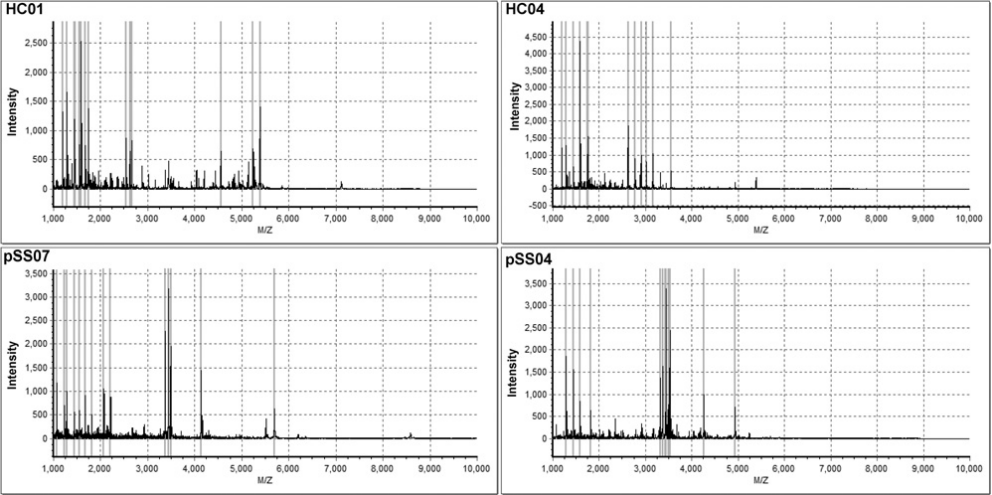
The entire mass spectra of the extracted peptide samples from two pSS patients and two healthy controls generated from the same instrument settings in the range from 1000 to 10000 Da

In the present study, WS samples from 7 pSS patients and eight healthy subjects were analysed and compared by the MS method. The mean and deviations of unstimulated saliva flow rates were 0.729±0.138 ml/15 min for the experimental group and 0.76±0.114 ml/15 min for the validation group. The general information of the subjects involved in this study was presented in Table 1. The entire mass spectra of the extracted peptide samples from 15 subjects in the two groups were generated using the same instrument settings in the range from 1000 to 10000 Da (Figure 1). Most peaks were detected in the range of 1000–3500 Da.

**Figure 2:**
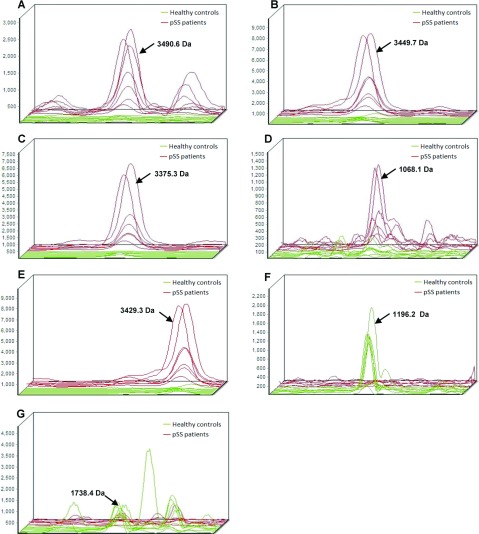
Three-dimensional m/z ratio-intensity maps showed the seven significantly different peptides at 1068.1, 1196.2, 1738.4, 3375.3, 3429.3, 3449.7 and 3490.6 Da Green curve, healthy control group; red curve, pSS group.

**Figure 3:**
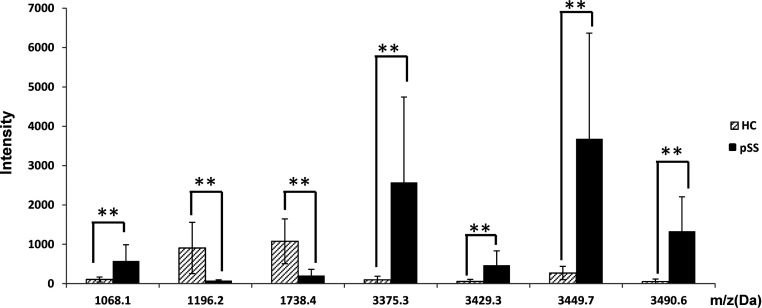
Column view of the mass spectra from the two groups, showed an increasing expression of peptides at 1068.1, 3375.3, 3429.3, 3449.7 and 3490.6 Da, and a decreasing expression at peak 1196.2 and 1738.4 Da in pSS patients

An average of 60 peptide mass peaks was found when the two groups was compared. Then the peaks among the mass spectra were quantified and compared. Seven of these peptide mass peaks (1068.1, 1196.2, 1738.4, 3375.3, 3429.3, 3449.7 and 3490.6 Da) were significantly different between SS patients and healthy controls (Figures 2 and 3). Among them, five peptides (1068.1, 3375.3, 3429.3, 3449.7 and 3490.6 Da) were up-regulated and two (1196.2 and 1738.4 Da) down-regulated in the pSS patients.

**Figure 4:**
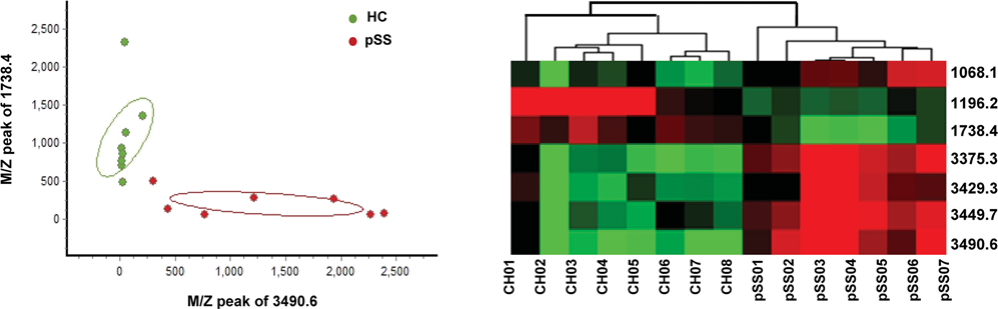
Scatter plots of the two groups established by combining peptides 1738.4 and 3490.6 Da, and the clustering analysis of seven peaks with significant difference (*P*<0.01, by *t* test) in their distribution between healthy controls and pSS patients

All of the seven mass peaks were used to establish the diagnostic model by the RBF (Radial Basis Function) method. The two mass peaks (1738.4 and 3490.6 Da) exhibited the most significant difference (*P*<0.01, by *t* test) when the two groups were compared, and the fitted results of the other combinations were not as good as it. Thus, we use these two peptides to establish a fitted curve. In addition, all of the seven mass peaks were used for clustering analysis (Figure 4). The arrangement the intensities of the seven peaks in 15 samples in binary format was by unsupervised, average-linkage hierarchical clustering using standard correlation as a distance metrics between the pSS group and the control group. Columns represent samples; rows are m/z peaks as indicated by the average molecular weight. The shape of the two figures showed the well-separated locations of the samples from the two groups, indicating that the fitting results were satisfactory.

Validation of the diagnostic model was carried out in a new series of five SS patients and five healthy controls. Four of the five SS patients were diagnosed as pSS and four of the healthy controls were diagnosed as healthy subjects, respectively, by this diagnostic model.

Moreover, the successfully identification of peptide at 1738.4 Da was predicted to be KNG1 Isoform LMW of Kininogen-1 precursor, which acts as a mediator of inflammation and causes increase in vascular permeability, stimulation of nociceptors and release of other mediators of inflammation. Peptides of 1068.1, 3449.7 and 3375.3 Da were predicted to be FGA Isoform 1 of Fibrinogen alpha chain precursor, which yields monomers that polymerize into fibrin and acting as a cofactor in platelet aggregation.

## DISCUSSION

pSS is an autoimmune disease characterized by progressive lymphocytic infiltration and destruction of the lachrymal and salivary glands, which leads to a marked reduction in tear and saliva secretion. Owing to its wide variety of non-specific symptoms, the condition is often overlooked or misinterpreted, and a definitive diagnosis is made an average of nine years post-onset [13]. This under-diagnosis negatively affects patients’ quality of life as it delays proper treatment. Therefore it is important to find a method of diagnosing early pSS.

In recent years, interest in saliva for clinical purposes as an alternative to other body fluids, such as blood and urine, has increased. WS is a complex biological fluid since several contributors are responsible for its production. In addition to the exocrine components, there are several non-exocrine contributors, such as desquamated epithelial cells, intact and partial blood cells, gingival fluid and possibly fluid entering the oral cavity through mucosal seepage. This renders diagnosis of disease by the analysis of saliva both challenging and attractive. MS-based proteomics is a high-throughput method used for the analysis of salivary proteomics and has been employed in the study of protein/peptide spectra, biological marker spectra, or single biological markers for complicated diseases, such as cardiovascular and cerebrovascular disease, tumors, SS and neuro-degenerative disease.

To-date, WS has been found to contain more than 1300 proteins of different origin, such as the salivary gland, plasma/serum, GCF (gingival crevicular fluid), minor glands and oral epithelia [14]. In a 2005 study, 309 proteins were identified in the whole salivary fluid of healthy subjects using proteomics. Ninety-two proteins/peptides below 3 kDa and 19 of 3–10 kDa were identified [15], comprising approximately 40–50% of the total secreted proteins [16]. Based on previous studies, salivary peptides are usually classified into five groups according to their intrinsic properties: PRPs, histatins, cystatins, statherin and defensins [17]. Salivary PRPs (as well as bPRPs (basic proline-rich proteins), aPRPs (acidic proline-rich proteins) and gPRPs (glycosylated proline-rich proteins) are usually identified from the small peptide fraction (<3 kDa). Some of the PRPs, along with statherin and histatin-1, appear to actively participate in tooth mineralization. Histatins, especially histatin-5, which is found in high amounts in saliva, are strongly antifungal [18]. The cystatin class comprises five major isoforms (S, C, D, SA and SN), which have strong bactericidal and virucidal properties [16]. Statherin, a multifunctional molecule that possesses a high affinity for calcium phosphate minerals such as hydroxyapatite, contributes to the maintenance of the appropriate mineral solution. Defensins are a family of low-molecular-weight (3–4 kDa) cationic proteins with antibiotic, antifungal and antiviral properties. They are involved not only in innate immunity against infections but also in adaptive immunity, inflammation and wound repair [19].

Study of early markers of primary SS is a hot topic of investigation around the world. Development of effective and non-invasive methods that can be applied to the diagnosis and treatment of pSS has become the focus of research. In 2007, Shen Hu et al. reported that the expression of 16 WS peptides was significantly different in pSS patients and controls. Ten of the 16 peptides were overexpressed and six were underexpressed [9]. Peluso carried out a proteomic study of salivary peptides and proteins in patients with SS before and after pilocarpine treatment [7]. They found that pSS patients were characterized by higher α-defensin 1 levels and by the presence of β-defensin 2, and that the basic and acidic PRP groups and the statherin group showed the best response to pilocarpine.

In our study, WCX magnetic beads and the MALDI-MS technique were employed to investigate WS samples from pSS patients and healthy controls. The components extracted by the WCX magnetic method in our study could be either low-molecular-weight peptides or fragments resulting from proteolytic activity occurring in the WS after secretion into the oral cavity. The effectiveness of this combination of techniques has been confirmed in many serum-based peptide profile identification studies [20,21]. In total, seven pSS saliva samples and eight healthy control samples were examined. Seven m/z peaks (m/z 1068.1, 1196.2, 1738.4, 3375.3, 3429.3, 3449.7 and 3490.6) were found to be significantly different between pSS patients and healthy subjects. Five of these were up-regulated (m/z 1068.1, 3375.3, 3429.3, 3449.7 and 3490.6), and two were down-regulated (m/z 1196.2 and 1738.4). We found that the mass peaks of 3375.3 and 3449.7 were detectable in all pSS samples at a high intensity, but in none of the healthy subjects, suggesting that these represent markers of pSS and may play a role in the occurrence and development of this disorder. The 1196.2 and 1738.4 mass peaks were detected in the majority of healthy subjects, but absent in pSS patients.

Our results also differ from those of Hu et al. in 2007. They found 16 significantly different peaks in pSS patients and healthy controls. Ten of these (m/z 1107, 1224, 1333, 1380, 1451, 1471, 1680, 1767, 1818, and 2039) were up-regulated and six (m/z 2534, 2915, 2953, 3311, 3930 and 4187) were down-regulated. The differences between the two studies may be attributable to the different age and race of the participants involved, and/or to the different methods used to extract low-molecular-weight peptides and proteins. The participants enrolled in Hu's study were all Caucasian women with a mean±S.D. age of 37.2±9.8 years for the pSS patients, and 37.0±10.6 years for the healthy control subjects, while our study used Chinese women with a mean±S.D. age of 53.9±12.26 years in pSS patients and 46.9±1.66 years in healthy controls. It is possible that the WS proteome changes with age [22]. However, it has not been proven that the saliva proteome differs between races. The small peptides (<10 kDa) used for MS analysis in our study were extracted from WS samples by WCX magnetic beads, while the saliva samples used in Hu's study were precipitated by cold ethanol followed by centrifugation, and the supernatants were collected and dried with a speed vacuum for peptide biomarker analysis. Nevertheless, the different processing methods could lead to artificial losses and modification of the samples, which could influence the results significantly [14].

In conclusion, the saliva proteome is an attractive prospect for the early diagnosis of SS using the MALDI-MS technique. This method could provide a new tool to diagnose SS rapidly and with low cost. However, further studies should be carried out using a larger number of patients and healthy controls so as to fully explore the clinical value of differentially expressed proteins as markers of this disease. Moreover, identification of the biomarkers will help establish a concise model for pSS diagnosis.
